# Reconceptualising public acceptability: A study of the ways people respond to policies aimed to reduce alcohol consumption

**DOI:** 10.1177/1363459315574117

**Published:** 2015-03-13

**Authors:** Simon Cohn

**Affiliations:** London School of Hygiene & Tropical Medicine, UK

**Keywords:** alcohol, focus groups, health behaviour, health policy

## Abstract

The issue of public acceptability of health policies is key if they are to have significant and lasting impact. This study, based on focus groups conducted in England, examines the ways people responded to, and made sense of, policy ideas aimed at reducing alcohol consumption. Although effective policies were supported in the abstract, specific proposals were consistently rejected because they were not thought to map onto the fundamental causes of excessive drinking, which was not attributed to alcohol itself but instead its cultural context. Rather than being influenced by the credibility of evidence, or assessed according to likely gains set against possible losses, such responses were established dynamically as people interacted with others to make sense of the topic. This has significant implications for policy-makers, suggesting that existing beliefs and knowledge need to be taken into account as potentially productive rather than obstructive resources.

As the World Health Organization (WHO, 2009) has stated, ‘Having a good policy is not enough: to be effective, policy requires public support’ (p. 2). The extent to which a policy might be acceptable is normally assessed through some kind of survey method designed to capture representative views and attitudes (Banerjee et al., 2010; Branson et al., 2012). Often, authors who go on to write about the acceptability of a specific government policy refer to existing public knowledge and preferences to make sense of such results (see, for example, Hackley et al., 2008). An article by Lonsdale et al. (2012) is a useful illustration; it provides insight into people’s views with respect to a minimum price per unit of alcohol policy based on focus group data. One of the key arguments the article goes on to make is that the findings indicate people do not believe a minimum unit price would reduce overall alcohol consumption, so they consequently recommend that if such a policy is ever introduced, it should be accompanied by ‘an educational campaign aimed at changing beliefs and attitudes’ to ‘dispel any misconceptions’ and hence increase its acceptability.

However, the degree to which people endorse a policy or find it acceptable is far from a straightforward matter, and is likely to be influenced not only by what they know and believe individually but also more general and emergent orientations in social settings. As such, while Lonsdale et al. are at pains to emphasise that their results are ‘transferable’ rather than ‘generalisable’, the methodological orientation of their study is one in which the focus group is viewed as providing access to the pre-existing views of the sampled participants. This is, of course, entirely defensible if one accepts the general premise that a person’s attitudes and beliefs are derived from a wide range of influences, for example, from public information and the perception of societal norms, but become stabilised and individually ‘held’ or stored. According to this position, a focus group provides an opportunity for them to be expressed and as a result can be accessed by the researchers. Yet, from another perspective, the approach could be said to reproduce ideas of public acceptability uncritically and, in tandem, overlook much of the literature in sociology on focus group studies which resists the idea that what people say in this forum can ever be reported uncritically (Carey and Smith, 1994; Duggleby, 2005; Morgan, 1996).

In contrast, therefore, this article adopts a broadly interactionist approach to examine the ways in which people respond and make sense of policy ideas related to alcohol consumption as they talk with others and establish common viewpoints. Rather than thought of as faithfully articulating pre-existing views or establishing opinion independently, such responses are considered the result of dynamic exchanges with others which serve to develop and consolidate a position. This orientation, therefore, challenges much of the acceptability research which frequently reifies the degree of acceptableness as a stable attitude associated with a particular issue. More specifically, this approach enables an exploration of two underlying logics present in much of this literature across many topics – that people establish a position based on an economic-like rationality (Wolfe et al., 2002) and that, as representatives of ‘the public’, individuals assess a policy not only in terms of how it might impact them personally but also how it might affect ‘the population’ in the abstract.

The issue of public acceptability of health-related policies is a case in point. In relation to the first logic, both policy-makers and researchers frame issues in terms of unavoidable trade-offs that have to be made between potential health gains and a variety of losses, such as freedom of choice. In addition, this cost–benefit paradigm lies beneath many related discussions, such as the ‘intervention ladder’ offered by the Nuffield Council of Bioethics which grades policies on the extent of their intrusion into people’s lives and hence the degree of negative impact that might need to be counterbalanced (Nuffield Council of Bioethics, 2007). As a consequence, policy-makers frequently emphasise the importance of disseminating relevant scientific evidence in a comprehensible way to ensure the public fully appreciate the potential health gains and overall ‘utility’ of any new intervention that will outweigh any perceived costs (Branson et al., 2012; Russell et al., 1996). However, in recent years, conceptualisations of the link between knowledge and public understanding have moved from simple deficit or diffusion models to ones that foreground the diversity of publics and the multiple ways in which knowledge is translated and emerges as meaningful (Horst and Michael, 2011; Lambert and Rose, 1996). For example, research has shown how health promotion campaigns are often negotiated in complex and contradictory ways, combining resistance, denial and attributing relevance only to other people (Thompson and Kumar, 2011). Recognising that knowledge ‘is always produced in, and part of, the context of local cultural conditions’ (Lidskog, 2008: 72), such perspectives challenge the idea that improving the understanding of evidence necessarily leads to the greater acceptability of a policy by ensuring an appreciation of the benefits to health will outweigh perceived costs.

The second key assumption is that people are able to differentially focus on the possible impact a policy might have on themselves compared to its wider public good (see Edwards, 1997). Although, of course, particular health policies can and do focus on segments of the population – for example, ones aimed to support vulnerable or marginal groups – the general emphasis of contemporary government is to try and address health-related behaviours of the whole population. One of the issues that policy-makers, therefore, assume needs addressing is how to ensure the ‘prevention paradox’ is not fore-grounded (i.e. that an intervention might have great benefit to the population but be negligible for the individual, see Rose, 1981). Some have argued that, as a result, non-experts adopt a kind of ‘lay epidemiology’, in which they make sense of health risks by drawing on cases of illness in their own social worlds (Frankel et al., 1991). For example, the much-described ‘Uncle Norman’ and ‘Last person’ responses (Davison et al., 1991) can be interpreted as strategies which actively disregard or resist policy rationale and its accompanying evidence-base by embedding instances of illness and health within a landscape of personal experience and local social networks rather than according to a distribution across an abstracted concept of ‘the population’.

This article addresses these two underlying assumptions in much of the policy acceptability literature – that people weigh-up potential benefits against harms and that they think about policy impact in terms of a notion of ‘the population’ – by foregrounding the intrinsically emergent nature of people’s views and assessments. Drawing on a simple set of ideas around attempts to reduce alcohol consumption, it describes how people respond to health behaviour policies and largely resist them, not according to an overt cost–benefit analysis or because the proposals are conceptualised in epidemiological terms, but rather through ongoing social processes of contextualisation and affirmation that ultimately reconceptualises both what the ‘the problem’ is and where it is located.

## Policy ideas to reduce alcohol consumption

Alcohol has always been intertwined with matters of politics; such things as taxation and licensing have meant consumption in the United Kingdom has been influenced by legal and political control for centuries, to the extent that these have become features that form an integral part of the meaning and experience of drinking practices (House of Commons Health Committee, 2010). But the issue of excessive alcohol consumption as a health problem and how the government might tackle it has never more been a contemporary issue – both in the United Kingdom and internationally. Such political and legal framing invariably treats alcohol beverages as discrete and singular – distinct from food and other drinks (see for example, O’Dowd, 2011). As a result, when it comes to policies to reduce excessive consumption, they tend to present alcohol, generically, as the ‘problem’. For example, a WHO (2009) report considers it to be ‘a special and hazardous commodity’ (p. 2), while a key Lancet publication categorically states that alcohol ‘… is a determinant of health that contributes to health inequalities’ (Casswell and Thamarangsi, 2009: 2247). Through this language, the risks to health and society come to be causally associated primarily with the physical substance rather than the many complexities that are known to determine drinking behaviour.

In contrast, social science research has tended to focus on everyday lived experiences and the extent to which drinking practices are conducted within a range of broader social contexts, which can include many individual and social benefits as well as harms (The Social Issues Research Centre, 1998). Initial writings on the cultural value of consuming alcohol tended to adopt a somewhat functional perspective (see, for example, Douglas, 1989), but as this work has been extended, more nuanced accounts have demonstrated the extent to which drinking practices not only consolidate but also demarcate different social groups, establishing various individual and social identities (Bucholz and Robins, 1989). One of the key difficulties policy-makers, therefore, face is the inherently ambivalent view of alcohol that emerges from these more sociological accounts; its consumption is both enjoyable and pathological, regarded as communally valuable yet also perceived to be socially damaging (Nicholls, 2010). In other words, any attempt to promote policies to limit consumption by simply emphasising the dangers of alcohol inevitably ignores the many pleasurable and positive dimensions that people value and might expect to be acknowledged (Szmigin et al., 2008).

Critics of government attempts to control drinking and the intersection ‘of individual behaviour with state regulation’ (Malleck, 2012: 8) have drawn on such concepts as biopower and governmentality to explore the ways policies are traditionally designed to both prohibit and promote certain kinds of subjectivities. It is argued that by targeting the individual, such policies are merely part of the more general shift towards ‘responsibilisation’ and the imperative that people should make healthy choices (Lupton, 1997), thereby failing to take into account wider structural forces and excluding matters relating to pleasure or spontaneity (Griffin et al., 2009). However, a recent feature of contemporary UK policy thinking has been to shift emphasis away from trying to educate people so that they might alter their behaviour voluntarily towards adopting more specific, instrumental approaches. Systematic reviews and meta-analyses have identified minimum unit price and controlling its availability as two highly cost-effective policy levers (Anderson et al., 2009). Notwithstanding legislative matters, these are relatively simple interventions designed to influence a complex social issue – not by re-fashioning individuals but through direct control of the environment. Yet, the public appears to be more sceptical of such strategies compared to other attempts to reduce alcohol consumption (WHO, 2009).

It is against this backdrop of current alcohol-related policies and the central importance placed on public acceptability that the article draws on focus group data to capture something of the processes that, in combination, might be said to constitute how such responses to policy ideas are established. While this method does not reproduce aspects of people’s everyday lives directly, analysing the processes of discussing, agreeing, disagreeing and developing a position provides insight into the ways that people come to establish a response to policy that is both individually and socially derived. Consequently, by examining how a policy idea comes to be talked about, the issue of acceptability is alternatively framed as a position arrived at through conversation with others rather than being an individually held view that can be retrieved by direct interrogation.

## Methods

The design of the study, including the use of visual and textual stimuli to trigger discussions, was intended to ensure that a range of views would be expressed rather than simply record a collective consensus (Barbour and Kitzinger, 1998). There has, of course, been a long-standing debate about the so-called gap between attitudes and behaviours (see LaPiere, 1934 for what is often identified as the first acknowledgement of this) which potentially divides those who use qualitative accounts assumed to correspond to what people objectively do and those who foreground the research process as one of co-production and the primary object of research, irrespective of whether this relates to everyday behaviour or not. Although this tension has always informed the social sciences, what is significant is the extent to which this can potentially generate a very different approach to ideas of public acceptability to much of the health literature, which tends to reproduce the idea that acceptability is the result of a set of pre-determined dispositions, attitudes and beliefs.

The overall approach of this study consequently rests on the assumption that how people understand and talk about an issue is contingent upon their interactions with others. An interactionist approach, therefore, is not merely one that simply emphasises the ways in which individuals present themselves and respond to others in particular social contexts (Barbour, 2008) but also offers potential to investigate the ongoing process of people giving, and making, accounts about things that they might not normally articulate (Potter and Wetherell, 1987). Thus, rather than adopt the stance commonly used by policy-makers and market researchers, in which focus groups are considered to ‘give voice’ to a particular segment of the population or elicit a pre-existing set of views, this study emphasises how talk is constitutive (Kitzinger, 1994), providing insight into some of the more complex and relational aspects of making sense of health policy issues. The point is that the artificial nature of a focus group – bringing together people who have never met each other before – nevertheless can provide a highly productive way to capture views as they are established through interaction. Although these positions are sometimes based on ones that have been expressed before, and they may go on to be repeated in future settings, what is crucial is that they are never entirely fixed or context-free because they always arise through the dynamics of a particular social setting. Thus, the methodological orientation of this study is that ‘public opinion’ is always an emergent quality as people interact with others to establish grounds of similarity and difference, and that this is especially relevant when trying to investigate aspects of people’s lives that are not frequently talked about.

A total of 12 focus groups lasting between 90 and 120 minutes were held, comprising of between 7 and 10 participants. Participants were recruited by a research agency using a purposeful sampling frame that selected demographically representative groups from inner and outer London using The Market Research Society’s (2006) occupational categories (see Table 1). They were reimbursed for travel and childcare costs and were paid to participate in accordance with the agency’s usual practice. All focus groups commenced with a standardised introduction to the research topic, followed by a word association warm-up exercise before introducing a range of trigger prompts. These real and fictional stimuli included a short video, a selection of photographs and other images, textual extracts from media and research sources and objects such as bottles with alcoholic content labelling. This multimedia assortment was adopted to ensure discussions were always lively and that participants remained engaged and enjoyed participating. Although all the groups followed the same general scheduling, specific prompts varied according to a pre-designed ‘road map’ that guided the facilitators along alternative sequences of topics in order to respond appropriately to the flow of each session. The core stimuli, identified during two pilot study groups as the most productive, were always presented although not necessarily in the same order. The facilitators aimed to encourage conversation and debate to develop between participants with minimal intervention rather than to seek clarification and segue into new topics when conversation flagged.

**Table 1.** Demographic characteristics of the focus group participants.

Total	94
Age range	19–68 years (mean: 39.5 years)
Gender	Male: 48; female: 46
Nationality	British: 88; Nigerian: 1; Jamaican: 1; Sierra Leonean: 1 (3 missing data)
Current employment	Full-time: 48; part-time: 10; self-employed: 4; student: 2; unemployed: 14; retired: 6 (10 missing data)
Ethnicity	White: 53; Back/Black Caribbean: 17; Mixed: 4; Asian: 3 (17 missing data)
Household income range	£8000–£250,000
Main source of income	Salary: 57; government benefits: 12; pension: 9; savings: 2 (14 missing data)
Home status	Owner occupier: 53; renters: 29; living with parents: 5 (7 missing data)
Alcohol consumption	Drinker: 70 (ranging from ‘everyday’, ‘about 15 units a week’ to ‘weekends only’ or ‘about a bottle of wine a month’); non-drinker: 21 (3 missing data)
In addition, 12 lived alone, 27 were current smokers and 11 described themselves as having an ongoing illness of some sort (such as asthma, arthritis and back pain)	

The focus groups were video and audio recorded; the audio was transcribed verbatim by an independent service and then checked using the video files. The data were first subjected to a free-read in order to assess the scope and depth of the material and generate broad thematic areas. Using NVivo primarily as a data management system rather than means to conduct analysis, sections of discussion relating to specific stimuli were initially coded and compared across the groups. As immersion in the data continued, these more descriptive codes were complemented by more analytical themes that emerged across the groups and frequently across the various prompted topics. In order to ensure that context was always taken into account, NVivo enabled every statement or utterance could be looked at in relation to proceeding and preceding discussion from other group members.

The study was approved by the Cambridge Psychology Research Ethics Committee, Council of the School of Biological Sciences, University of Cambridge. Participants were able to choose their identity or were allocated pseudonyms.

## Results

### Redefining the problem: ‘I don’t think it’s the drink itself’

Rather than talk about a policy ‘in the abstract’ – for example, the suggestion that there should be a minimum unit price, or that supermarkets should not be able to promote buy-one-get-one-free offers – participants employed common ways to talk about the topics by drawing on situated accounts and illustrations. Overall, while people generally understood the logic of proposals that aimed to reduce consumption by controlling price or availability, not one of the participants expressed support for these approaches; although the rationale of these policy ideas were not necessarily denied, they nevertheless were resisted. Such interventions around unit price and availability were felt to be indiscriminate and inappropriate – not merely in terms of who they affected but critically because they ignored the different times and places in which drinking is done and the diverse underlying reasons.

An additional common feature that emerged during every focus group was the way that, as discussion unfolded, a clear distinction was made between the behaviour of drinking and the substance of alcohol. This enabled a range of different responses and reactions to policy ideas to include a far wider range of values and concerns. For example, in the following extract, the discussion separates policies controlling alcohol with that of problem drinking. One person initiates discussion by saying that the UK Labour government tried to introduce a ‘continental café culture’ when it changed the licensing hours. He goes on to recount a particular, second-hand, anecdote to illustrate that although young people might be embracing increased access to alcohol, they are nevertheless drinking without eating, which is the opposite of anything that might be associated with a café culture:

… an older person like myself went to a young person’s bar and saw two girls come in and order a bottle of red and a bottle of white. And the person said to me ‘and they didn’t have any food’ and I said ‘well … of course they didn’t …’

Another participant responds by saying there is more to the problems associated with alcohol than whether drink is freely available or not. He goes on to make a distinction between British and Polish culture and suggests that something such as levels of violence cannot be directly related to the amount of alcohol consumed:

I definitely think there’s something else to it … because there are many cultures that they drink quite a lot in and they don’t have nearly as much violence or anything as we do here. So I don’t think it’s the drink itself … For example when I went to Poland, they drink like crazy but they’re not violent, I didn’t get that vibe at all.

A third member of the group draws on yet another cultural generalisation, this time the French, to support and build on the common view:

… the French kind of drink wine with meals and stuff so the children in the families are getting that exposure that, you know, you can drink alcohol sensibly in a civilised manner, it doesn’t have to be all crammed into 48 hours over a weekend …

As the discussion progresses, others add their own experiences and anecdotes, so that the topic shifts from the initial one about levels of alcohol consumed to the idea that the problem is culturally specific. A similar progression occurred in all the focus groups, usually culminating in a discussion about educating ‘young people’ or ‘the next generation’; as one person expressed it, ‘Yeah, the government should do more than just increasing the price, educating people, the effects of alcohol’. This point is then picked up by another and further consolidated: ‘Culture change. Education and then tell people how to drink, don’t make drink as an evil thing …’

Making cultural generalisations and comparisons provided a frequent way for people to talk about consumption and illustrate how a focus on alcohol itself, as a singular and common element, was largely irrelevant. Rather than assume these cross-cultural references are crude or uninformed attempts to provide counter-evidence to a policy, they instead provide a more open ‘site of action’ for ongoing dialogue with others. In doing so, such repeated references to ‘other’ social contexts, whether in terms of different cultures or historical periods, rapidly established a sense of solidarity with others in the group, even though they had never met each other before. In doing so, the central problem is re-framed; any link between price and availability with changes in behaviour is dismissed for ignoring the role of culture and context in determining whether or not drinking alcohol is a problem. And crucially, the various harms and benefits of consuming alcohol become fully entangled with a wider set of values and cultural association that largely resist calculation.

### Collective imagination: ‘Our binge drinking’

The general position agreed upon – that what was required was a change in culture – served as a response not only to suggest the policies designed to control alcohol would be ineffectual but that demonising alcohol, as Mo above refers to, is, in a very real sense, perceived to be a criticism and attack on everyone – on ‘British culture’. This sense of the collective not only united the people in the focus groups but also underscored the sense that they were quite willing to consider themselves members of a social whole in relation to policies and the government, both of which they viewed as external. In another section of talk a participant raises the issue of ‘binge drinking’ alongside the words ‘we’ and ‘our’ not to suggest she sees herself as a binge drinker but rather that as a member of society she feels it is a shared problem to solve:

I think, it’s more making a point that alcohol is fun, that’s never going to go away, we all like a drink but there’s certain levels where you need to stop. And our binge drinking, I think, is our problem and I think it’s tackling that rather than a cost on a pint.

This comment is reiterated by another speaker who contrasts Cuba with England to suggest excessive ‘binge’ drinking is specifically an English problem:

You see, I travel immensely all over the world and I’ve gone to many Communist countries … like Cuba. They got plenty of drinks as well even though poor but I don’t see any drunk people on the street or anything. This is more of a culture in England that people have to indulge.

Although the spontaneous adoption of the term ‘binge drinking’ reflects a general acceptance of government and media portrayals associated with youth culture, a younger participant questions this. He asks whether overt and visible binge drinking is the main problem or whether the main problem is the overall level of drinking within society. The first speaker reaffirms a strong distinction between overall amounts of alcohol consumed, and ‘binge drinking’, which she says is the real problem. In this and other exchanges, not only the idea but also the very word ‘binge’ serves as a marker for something associated specifically with British culture. As another speaker expressed,

… the French people is more like home drinkers, they’re not like the British go in the bar, go in the restaurant and drink … they drink and have something to eat in a family or friends environment. Here, it’s a trophy if you’re five pints. How many? ‘Oh, I had fifteen pints last night, mate …’

Similarly, in the following excerpt, the phrase ‘Booze Britain’, a common alliteration used in the national media and also by UK politicians, confirms the idea that binging and its associated antisocial behaviours is now regarded by some as an embedded feature of contemporary society as a whole, as much an activity practised by a particular group. What is interesting in the progression of this topic is how identifying binge drinking with specific, ‘other’ groups gradually shifts to a more inclusive association with British society. As one says, ‘… it’s sort of accepted that people just go out and get hammered and have a fight in the street that that’s sort of okay behaviour’. This point is picked up by another person who says it is not even necessary to ‘go too far out’ to highlight how culturally specific the problem in the United Kingdom is. He goes on:

I went to Berlin and … there’s no people on the street going all crazy … they’re just cool and very respectful even if they’ve had a drink and they’re a bit wavy, you know … So I definitely think it’s not the drink that is the problem, it’s something else …

The reference to ‘bad’ behaviour is contrasted here with the word ‘respectful’. As the discussion develops, one person concludes that ‘it’s the culture that is the real problem’. Not only does everyone around the table vociferously agree, but by doing so, they see themselves as opposing how the government views the problem.

The common trajectory of discussion in all the focus groups was that, as the participants talked, policy ideas centring on price and availability increasingly came to be viewed as misplaced and that the government was ‘out of touch’ and failed to grasp what the real problem was. The examples show that the use of cultural comparisons not only reiterates the idea that if the specifics of British culture are not addressed, policies are unlikely to have any effect, but they also shift focus from the health risks of the individual onto a discussion of the well-being of the society more generally. Accounts of ‘other’ cultures foster a transient sense of solidarity within the group, as they add to and embellish each other’s positions. But in addition, this was underscored by an agreed sense that the UK government really had no idea about people’s lives, whether this related specifically to people who might exhibit problematic drinking behaviour or the lives of the people sitting around the table in the actual focus groups.

Significantly, no one in any of the discussions made definitive distinctions between themselves personally and British, or English, society. As frequently people would shift between them within a short section of talk, they did not view themselves solely as ‘an individual’ in the abstract but drew on being member of an imagined collective. For example, when talking about ‘binging’, speakers regularly switched from talking about this in terms of specific groups (namely, young adults) to it being a shared problem. In this way, what policy-makers might assume about how people negotiate the relationship between themselves and population, the extent to which they make demarcations between a policy affecting them personally and other people and overall how levels of public acceptability is arrived at fails to take into account the dynamic way in which people continually associate and disassociate with each other.

### Ineffective and counterproductive: ‘People just start making their own’

The examples above illustrate how policies were talked about in terms of them failing to attend to the real problem and, as a result, came to be described as ineffective, reflecting the commonly held belief that effective solutions reflect the provenance of a problem. Central to this is the way cross-cultural comparisons are regularly drawn upon to highlight the unique nature of the British context. However, at other times, in the discussion, cultural comparisons are instead used to establish universal accounts of how people react to government interventions. For example, one person compares the United Kingdom and the Middle East, where she says people are driven ‘underground’ to behave in secretive and surreptitious ways, saying, ‘If you look at countries where there are really, really strict regulations … they have to hide and sneak and get just as drunk as you do on the …’. At this point, another person joins in with a similar account as a general consensual view is reached:

… you’ll get to a stage where alcohol and cigarettes become, you know, black market stuff … I have a friend who lived in Doha, which is Arabic country, and it’s a completely dry country, no drinking … the locals on the outside, don’t drink alcohol and don’t do this, don’t do that … but behind closed doors they’re getting rat-arsed like the rest of … and they just sell it from a hole in the wall somewhere, just behind the government’s eyes.

The underlying theme established is that if people really want to do something, there is nothing the government can do to stop them; after some discussion, one participant provides the closing statement that it is ‘because as a culture it’s almost suppressed’. The reiteration of different national examples is in these instances used to evoke similarity rather than difference and that, therefore, behaviour will always be uninfluenced by the environment. On some occasions, this universalism is based on an idea of ‘human nature’, that is, that people are naturally compelled to enjoy alcohol and that as a consequence, some individuals will inevitably develop a drinking problem.

However, these more reductionist arguments frequently were picked up by another participants and embedded in accounts that constructed more contextual positions. For example, during a discussion of the common strategy of people ‘pre-loading’ (in the United States, this is known as ‘pre-gaming’ and in Canada, ‘pre-drinking’) as a strategy to avoid the high price of alcohol in bars and clubs, one person proposes that people will always find a way to drink alcohol if they want to but supplemented this with the idea that getting drunk can provide a social and psychological release:

People want to get drunk … it’s one of these things, we do it on the weekends to escape the shit we went through Monday to Friday … people are drinking more now because life’s getting harder …

Similar to a number of other participants, she presents contemporary life as one in which things are getting harder, and that the desire to get drunk is an inevitable consequence of social life, rather than ‘human nature’ – perhaps even an entitlement. The argument that people will always drink, whatever a government tries to do about it, was frequently developed to claim that restrictions frequently lead to rebellion, more risky kinds of consumption, and are ultimately counterproductive. She concludes by stating that ‘… no matter what you do people will find a way to get drunk, even if it means they’ve got to start drinking petrol with a bit of lemon juice in it’. Similarly, another describes how young people unable to purchase alcohol simply take up an alternative:

There’s another side effect, is that some of the youth market will be tempted towards drugs which then become much cheaper than the alcohol […] when I was eighteen I lived in America for six months and everyone in our sort of student house was smoking marijuana and things like this […].

He goes on to suggest that people everywhere are principally the same and that one can, and should, learn from what has happened elsewhere and, uninterrupted for a few minutes, returns to draw on other examples to emphasise his general point:

… this new drug in Russia … it’s been nicknamed Crocodile … it’s very cheap heroin basically so as the price for heroin has increased with the Afghanistan war, these addicts have been making their own drug. Now there’s a danger with alcohol as we’ve seen in India … where you have very poor population and the price goes up, they make their own one and a hundred and six of them died the other day because it was poisoned, you know. What I’m saying is, when prices change, people sometimes go other places, homemade or into more dangerous things.

This extensive listing of examples has the effect of producing a general warning that if the British government is too dictatorial, people if they feel constrained inversely respond in creative, and sometimes risky, ways. Similar points were developed by others using various historical rather than cross-cultural comparisons. In particular, alcohol prohibition was commonly mentioned:

I immediately think of the prohibition, they did similar and it just didn’t work did it, everything went underground and people still found a way to make their own booze and drink it.

Prohibition in the United States and minimum unit pricing were frequently paired together as examples of punitive and invasive state interventions that are likely to lead to similar social responses, as in the following exchange:

… any attempt to kind of reduce availability, whether it be through kind of increasing the cost … as they tried in America through prohibition, it doesn’t actually stop drinking. You kind of create, you know, people would be home brewing and stuff like that, you know, creating meths at home, you know.

The economic rationale of price and availability to reduce alcohol consumption is equated with cultural, religious and legal examples of restriction, evoking ideas of repression and release at both the individual and social levels. While people viewed work-time as a legitimate period for employers, and by extension for the government, to regulate and control their lives, alcohol was felt to be symbolic of both time and activity that should not fall within such jurisdiction. Shaped by ideas of escape and liberation, leisure activities were, in contrast, associated with notions of freedom and lack of restrictions. Even people who professed not to drink, for example, because of their religion, were similarly protective, recognising that consuming alcohol stood for a far wider set of ideas about leisure, relaxation and being able to choose. Thus, not only consuming alcohol but also the very principles of liberty were framed according to context.

## Discussion

The findings illustrate how the relationship between policy ideas to reduce alcohol consumption and the ways in which people respond to them is actively negotiated as they make sense of the topic. Using excerpts to exemplify interactions, they illustrate how, in the very process of talking about policy ideas, positions and arguments come to be constructed collectively. Through such crafting, the views on the policies are constantly assessed and re-formulated through an interactive process that draws on an ever-widening range of values and concerns rather than being based on individually held beliefs or deliberative evaluation. Core to this was the invariable shift in discussions from the substance of alcohol to behaviours of drinking. As speakers exchanged views and followed up the arguments of others, excessive consumption came to be presented as an intrinsically cultural rather than individual problem derived from the alcohol itself, and that governmental policies were, therefore, over-simplistic by trying to control one through the control of the other. As a result, the data suggest that acceptability is not a singular view based on the credibility of evidence, the calculation of likely gains set against possible losses, or any simple evaluation of who might benefit or be targeted within a population, but rather is a dynamic process that emerges through talking about and contextualising a policy within a specific social setting.

More specifically, this article highlights the common adoption of cultural comparisons which were regularly drawn on to resist policy ideas felt to mistakenly focus on the substance of alcohol itself rather than the social world in which people drink. Drawing on accounts of other cultures, and sometimes other historical eras, proved to be a particularly effective way to establish a conceptual distinction between drinking behaviour and drink. The views expressed by participants of drinking behaviour in different cultures served as mutually developed narrative accounts that, although not grounded in evidence, were strategically meaningful in relation to the policy ideas being discussed. However, these were not employed to refute one knowledge claim with another; in other words, they were not used as definitive sources of counter ‘evidence’ that an outsider might then try and evaluate as correct or not. Rather, they provided ways to talk through a topic with others in order for a collective orientation to be established. Presenting anecdotes in order to establish cultural contrasts enabled people to not only build up certain kinds of claims but also evoke values and emotional responses that were just as cogent as anything that an outsider might identify as ‘evidence’. The suggestion is not that making cultural comparisons is necessarily a feature of all talk about health policies but that these elements of speech, which were spontaneously adopted in this instance, reflect a more general process because they enabled a wide range of values to be aired. As a result, the specific policy ideas of controlling the price of alcohol, adopting a minimum unit price, or regulating its availability were made meaningless and inappropriate.

As Habermas (1989) has pointed out, the notion of the public sphere is itself culturally and historically derived and arose when members of the bourgeoisie formed a representative coherent body with which to voice their collective needs while maintaining private autonomy. According to Habermas, since this emergence of a liberal model of ‘the public’ during the 18th century, it has increasingly lost its coherence and instead become a sphere for competing claims and conflict, as capitalism increasingly blurs any distinction between private and public. Thus, rather than assume that the public constitutes a discrete and uniform sphere, and hence that ‘public acceptability’ can ever meaningfully refer to a consensual response to the government, this article illustrates how views emerge and are consolidated through specific social interactions. While the claim therefore means that a focus group can not be interpreted as directly illustrative of the ways people make sense of policies in their everyday lives, being able to capture some of the social processes nevertheless reveals the way in which the acceptability of health policy ideas are evaluated as an ongoing and interactive process. This suggests that the relevant context which makes a government intervention meaningful is not only much wider than policy-makers and survey researchers might sometimes assume, but is also one that is creatively established by individuals through their interactions with others.

Two underlying assumptions in much of the acceptability literature were highlighted at the beginning: that people are likely to assess policies in terms of trade-offs and that they frequently distinguish between how a policy affects them personally from how they imagine it might influence the population in general. However, neither of these two features surfaced during the group discussions as people worked through their responses. Of course, the notion of ‘homo economicus’ and the assumption that people are inherently driven to make rational choices in order to maximise their gain have been long criticised by social scientists for its simplistic understanding of how people make decisions in their everyday lives. The very varied considerations – for example, whether people find it reasonable, justifiable, tolerable, understandable and so forth – are unlikely to be marshalled by any common conceptual currency such that a summative calculation can ever be undertaken (see Le Grand, 1997). The point, therefore, is not to crudely suggest that people are inherently irrational or illogical, or that they are not able to conceptualise certain aspects of a policy in terms of potential costs and benefits or according to the overall effects on the population. Rather, the idea that factors can always be directly compared to each other in some calculable way in order to establish a single, overall position is more a theoretical assumption than a reflection of how people themselves come to regard whether a policy is acceptable or not. In addition, ignoring the more intangible, symbolic and affective attributes people often associate with particular factors means that the wide variety of conflicts and tensions that potentially confront people when they consider how a proposal might affect them in their everyday lives is frequently omitted by health researchers. While epidemiological approaches attribute risks to individuals as a consequence of an overall assessment of the population, it seems clear that members of the public frequently draw on accounts of society and culture that simply do not equate with such an orientation. As a consequence, there might well be no ‘prevention paradox’ to a policy. Instead, using specific anecdotes to conceive of the social – for example, through making comparison with other cultures – means that any abstract argument about total health gains is rendered meaningless by its very lack of situatedness.

The analysis described ways in which people resisted price-based policy ideas to reduce alcohol consumption and often discounted any suggestion of supporting scientific research of their potential effectiveness. Instead, they talked about the value of health education, in particular of the next generation, as both being more ‘fair’ and more ‘effective’. From a psychological perspective, this might be conceptualised as a self-serving bias – the desire not to pay more for alcohol driving a search for evidence to support the status quo. However, rather than frame this in terms of a rational strategy to counter one kind of evidence with another, what has been emphasised is the way that this response emerges from the interactions of the group rather than individual positions, and that the use of different accounts and anecdotes are used to incorporate qualitatively different kinds of knowledge claims. Adopting an anthropological perspective, Hansis (1996) proposes that policy acceptability is as much based on its interface with diffuse cultural values as much as any rational calculation of costs and benefits; in sum, he states that ‘to be acceptable, a new idea has to have meaning’ (p. 40). Such a comment implies that views on acceptability are likely to be drawn from both facts *and* values. But one should perhaps extend this argument; in practice, people rarely make such a distinction since both facts and values are forms of knowledge made meaningful in social life. As a result, any ‘meaning’ of a particular policy is likely to be derived from a wide range of considerations that are not necessarily consistent with each other and frequently not amenable to a common comparison. More than this, they frequently emerge in social context – even one as artificial as a focus group – since such a range of beliefs and concerns only manifest during social interaction, as ideas emerge, develop, are built on and are possibly stabilised.

The implications for policy-makers are that what is commonly talked about as levels of public acceptance already implicitly conceptualises the ways in which people respond to and make sense of a policy. For example, if public acceptability is understood to be low, a frequent strategy is to provide further supporting evidence in order to encourage debate and potentially shift opinion. There is some evidence to show greater public acceptance of an increase in alcohol taxation when the funds are earmarked for addiction prevention and treatment services (Tobin et al., 2011; Wagenaar et al., 2010). However, framed like this, such studies indirectly reproduce the idea that people can and do think in terms of weighing costs against benefits and that they primarily arrive at an opinion individually. Conversely, by acknowledging the extent to which social interactions serve to make sense of and establish a viewpoint collectively – something that might be akin to forging a ‘public’ opinion – suggests the notion of policy acceptability is likely to be far more of an ongoing engagement rather than a one-off assessment, and emerges as much from encounters with other people and other viewpoints as a position arrived at through individual reflection.
